# The Buffering Effect of Caregiver Education on Early Childhood Development in Low-Income Households: Evidence from Indonesia

**DOI:** 10.3390/children13060767

**Published:** 2026-06-01

**Authors:** Yuri Nurdiantami, Hilda Meriyandah, Tokie Anme

**Affiliations:** 1 Graduate School of Comprehensive Human Sciences, University of Tsukuba, Tsukuba 305-8577, Japan; s2236041@u.tsukuba.ac.jp; 2 Faculty of Health Sciences, Universitas Pembangunan Nasional Veteran Jakarta, Jakarta 12450, Indonesia; 3 Faculty of Medicine, University of Tsukuba, Tsukuba 305-8575, Japan

**Keywords:** socioeconomic status, early childhood development, child-rearing environment, Indonesia

## Abstract

**Highlights:**

**What are the main findings?**

- A family’s financial resources and a caregiver’s educational background independently shape the physical setup of the home environment, with neither fully replacing the other.
- For early childhood developmental milestones, higher caregiver education was associated with reduced income-related developmental differences among children in low-income households.

**What are the implications of the main findings?**

- Financial and educational resources may contribute to early childhood outcomes through different pathways: income may support material conditions, while caregiver education may be linked to caregiving knowledge and interaction quality.
- Future intervention studies should examine whether combining economic support with caregiver-focused guidance improves children’s developmental outcomes among low-income families.

**Abstract:**

**Background/Objectives**: Household poverty is a known risk factor for early childhood development. However, the extent to which caregiver education may moderate income-related developmental disadvantages remains underexplored in Southeast Asian contexts. This study investigated whether caregiver educational attainment buffers the association between household income, child-rearing environments, and early developmental outcomes in Indonesia. **Methods**: This study utilized cross-sectional data from Indonesian caregivers. To maximize statistical power, analyses of the home environment using the Index of Child Care Environment included the full sample (*N* = 933). Analyses of developmental outcomes using the Early Childhood Development Index were restricted to 3- and 4-year-old children (*N* = 355). General Linear Models (GLMs) were conducted, controlling for child age and sex. **Results**: For the home environment, both household income (*p* = 0.042) and caregiver education (*p* = 0.021) were independent, significant predictors, with no significant interaction. However, for developmental outcomes, the Income × Caregiver Education interaction was statistically significant (*p* < 0.001). **Conclusions**: Income and caregiver education were independently associated with the home environment, while their interaction was associated with developmental outcomes among children aged 3 to 4 years. These findings are consistent with an educational buffering hypothesis and warrant further longitudinal research.

## 1. Introduction

The intersection of domestic poverty and early childhood development presents a significant challenge in global public health and developmental science. Millions of children in low- and middle-income countries (LMICs) are at risk of not reaching their developmental potential due to systemic material deprivation [1]. The early years of life are characterized by rapid neurobiological and psychological maturation, establishing developmental trajectories that often persist into adulthood [2,3]. During this critical period, poverty acts as a pervasive environmental stressor, compromising both the physical resources within a household and the quality of the caregiving environment [4,5].

The Nurturing Care Framework and the Bioecological Model of Human Development are two theoretical frameworks that emphasize the significance of continuous, reciprocal interactions between children and their primary caregivers [6,7]. These proximal processes are heavily influenced by the family’s psychosocial and material resources. For example, in Indonesia, rapid economic development has been accompanied by persistent income inequality, which may increase developmental vulnerability among children in socioeconomically disadvantaged households. This makes many young children more vulnerable to the negative effects of poverty on their development [8].

Caregiver education may influence child development not only through economic resources but also through caregiving knowledge, communication, and daily parenting practices [9,10]. Caregiver education has been linked to richer language input, more responsive parenting, and greater investment in child-rearing resources. However, its role in moderating the association between low income and child development remains insufficiently understood, particularly in LMICs [11,12].

The Indonesian government has actively expanded early childhood education and development (ECED), yet coverage and quality remain uneven across regions and socioeconomic groups [13]. Moreover, evidence from national surveys in Indonesia shows pronounced socioeconomic inequalities in children’s cognitive performance and health, with poorer households experiencing lower cognitive scores [14]. Although parenting education programs are available, particularly in poorer communities, evidence remains limited on how caregiver education shapes the association between poverty and child development [15].

In Indonesia, many low-income families have some access to ECED services, but caregiver education may influence service choice, attendance consistency, and the extent to which learning is reinforced at home [13,16]. A buffering interpretation also fits the broader evidence base: caregiver education has been linked to better child growth and home-learning environments in LMICs, and studies in low-income settings show that educated caregivers are more likely to recognize needs, use services, and provide supportive care, which can weaken the usual poverty gradient in child outcomes [17]. Based on this evidence, we hypothesized that the association between low household income and child development would be weaker among children whose caregivers had higher levels of education [18,19].

Previous studies have often examined socioeconomic disadvantage, the home environment, and child development together, but fewer have distinguished how household income and caregiver education may relate differently to caregiving conditions and developmental milestones. This study addresses this gap by examining their separate and interactive associations in Indonesia [20,21].

This study examines whether caregiver education buffers the association between household income, child-rearing environments, and early developmental outcomes in Indonesia. More specifically, it examines how household income and caregiver education are each related to the child-rearing environment and child development, and whether the association between income and child development differs by caregiver education level. Drawing on prior work linking poverty to constrained material investments and caregiver education to more stimulating and responsive parenting, we hypothesized that the association between low household income and child development would be weaker among children whose caregivers had higher levels of education.

## 2. Materials and Methods

### 2.1. Study Design and Participants

This study represents a secondary analysis of a primary cross-sectional dataset originally collected to examine geographic variations in Indonesian child-rearing environments [22]. Rather than focusing on geographic location, the present study repurposes the data to track socioeconomic gradients. We shifted the analytical focus to treat household income and caregiver educational attainment as the core predictors.

We sampled participants from three distinct Indonesian settings with varying socioeconomic profiles: an urban environment (J city), a suburban area (D city), and a rural region (G Regency) between July 2023 and June 2024. Purposive cluster sampling was used for data collection. Clusters were defined as kindergartens and integrated public health centers (Posyandu) in the study areas. Because we used purposive cluster sampling of kindergartens and public health centers rather than simple random sampling, our data best represent caregivers of preschool children from households attending similar facilities in the study regions, rather than all Indonesian caregivers.

At each participating site, staff helped identify eligible caregivers and invited them to take part in the study after routine services or parent meetings. Self-administered questionnaires were distributed through local kindergartens and public health centers to reach caregivers in the participating communities. All data were collected using a self-administered electronic questionnaire accessed via tablet or smartphone. To minimize missing data, all items required a response before the questionnaire could be submitted. Only fully completed questionnaires were included in the analysis, and there was no item-level missing data for the variables used in this study.

The target population comprised primary caregivers of preschool-aged children. Caregivers were eligible if they were at least 19 years old and the primary caregiver of a child aged 3–6 years enrolled at one of the participating kindergartens or registered at an integrated public health center. The primary caregiver was defined as the adult identified by the household as having the primary responsibility for the child’s daily care. This person could be the child’s mother, father, grandparent, or another adult relative; when more than one adult was present, the caregiver most involved in day-to-day care was invited to respond.

Children with caregiver-reported diagnosed disabilities or chronic conditions likely to affect development, such as cancer, autism spectrum disorder, or attention-deficit/hyperactivity disorder, were excluded. Because participating sites did not record the number of eligible caregivers invited, a formal response rate could not be calculated, and potential selection bias cannot be excluded. The minimum sample size, estimated using Cochran’s formula with a 95% confidence level, 5% margin of error, and expected prevalence of 0.5, was 385 participants. The child-rearing environment analysis included 933 caregivers, while early child development analyses were restricted to 355 caregivers of children aged 36–59 months [21], consistent with the validated Early Childhood Development Index (ECDI) age range. Therefore, ECDI estimates should be interpreted as less precise than those based on the full sample. Institutional review board approval was obtained, and written informed consent was secured before participation.

### 2.2. Measurement

To assess the quality of the home environment, we used the Index of Child Care Environment (ICCE). The ICCE was originally developed in Japan as a brief parent-report measure of the quality of the home child-rearing environment and has shown good validity and reliability in previous studies. For this study, we translated the ICCE into Bahasa Indonesia using a forward–backward translation procedure, followed by expert review and pilot testing to ensure clarity and cultural relevance. Internal consistency for the 13 items in our sample was acceptable (Cronbach’s alpha = 0.778).

Parents completed this 13-item questionnaire by answering multiple-choice questions across four specific areas: The Human Stimulation subscale (5 items) captures the frequency of direct, reciprocal caregiver–child interactions, such as shared reading, face-to-face play, singing, and eating meals together. The Social Stimulation subscale (3 items) gauges the child’s exposure to external environments and social networks, including park visits, shopping trips, and interactions with similar-aged peers. To evaluate parenting style and disciplinary practices, the Avoidance of Restriction subscale (2 items) assesses the caregiver’s response to purposeful misbehavior (e.g., spilling a drink) and their recent use of physical discipline. Finally, the Social Support domain (3 items) examines the caregiver’s practical and psychosocial safety net by measuring their access to alternative childcare assistance and opportunities to discuss child-rearing with a spouse or external confidants. By summing the values of all 13 items, we calculated a final score ranging from 0 to 13. In this scoring system, a higher score indicates a more supportive home-rearing setting.

Developmental status was measured using the 10-item ECDI, developed by UNICEF and incorporated into the Multiple Indicator Cluster Surveys to monitor early childhood development at the population level in LMICs. The ECDI asks caregivers whether their child has achieved specific age-appropriate milestones across four domains: literacy–numeracy, learning, physical development, and social–emotional development. We used the Bahasa Indonesia version employed in national surveys to ensure conceptual and linguistic equivalence in the Indonesian context.

Following UNICEF guidance [23], each item was coded as 1 if the caregiver reported that the child had achieved the milestone and 0 otherwise. In addition, we derived the standard dichotomous indicator of being “developmentally on track” (on track in at least three of the four domains) and summed the number of domains in which the child was on track (range 0–4), with higher values indicating more developmental domains on track.

### 2.3. Statistical Analysis

Univariate General Linear Models were used to examine the main and interaction effects of household income and caregiver education on two continuous outcomes: total ICCE score and overall ECDI score. Household income and caregiver education were entered as categorical predictors, and child age and child sex were included as adjustment variables. The primary interaction term was Income × Caregiver Education, which was used to test the hypothesized buffering effect. Statistical significance was set at *p* < 0.05. Significant interactions were decomposed using estimated marginal means with Bonferroni-adjusted pairwise comparisons across income groups within each caregiver education level. Household income was reported as monthly income in Indonesian rupiah and categorized as low, middle, or high based on the Indonesian national average salary [24]. Caregiver education was recorded as the highest level of formal education completed and recoded into three ordered categories: less than high school, high school, and more than high school. All analyses were conducted using IBM SPSS Statistics ver. 30.0.

## 3. Results

Table 1 presents the sociodemographic characteristics of both the full study sample (*N* = 933) and the subset restricted to 3- and 4-year-old children (*N* = 355). In the full sample, the sex distribution was roughly equal, comprising 50.2% girls and 49.8% boys. The majority of children were 5 years old (47.6%), followed by 4-year-olds (29.4%). Regarding socioeconomic status, a significant portion of households fell into either the low-income (40.9%) or high-income (42.2%) brackets, with only 16.8% classified as middle-income. Most caregivers were mothers (94.1%), followed by fathers (3.4%) and other caregivers (2.5%). Caregivers’ educational attainment was predominantly at the high school level (50.9%), with 35.2% having more than a high school education and 13.9% not finishing high school. The restricted subset of 3- and 4-year-olds showed a broadly comparable demographic profile to the full sample across child sex, household income, caregiver–child relationship, and caregiver education level.

Table 2 presents descriptive means for ICCE and ECDI scores by household income and caregiver education. Mean ICCE scores were higher in the high-income group than in the low-income group, and were also higher among caregivers with more than a high school education. ECDI scores showed smaller descriptive differences across income and caregiver education categories.

Table 3 presents the GLM results for ICCE and ECDI scores. In the model for ICCE scores, household income, F(2, 922) = 3.192, *p* = 0.042, ηp^2^ = 0.007, and caregiver education, F(2, 922) = 3.877, *p* = 0.021, ηp^2^ = 0.008, were significant predictors after adjusting for child age and sex. Child sex, F(1, 922) = 0.006, *p* = 0.937, ηp^2^ = 0.000, and child age, F(1, 922) = 0.513, *p* = 0.474, ηp^2^ = 0.001, were not significant predictors. The Income × Caregiver Education interaction was not significant, F(4, 922) = 0.994, *p* = 0.410, ηp^2^ = 0.002, indicating that the association between household income and ICCE scores did not differ significantly across caregiver education levels.

**Figure 1:**
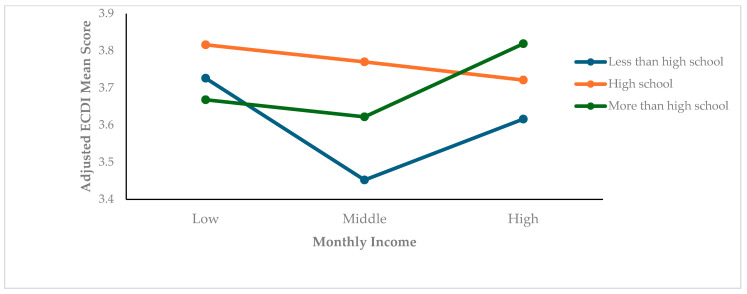
Adjusted ECDI mean scores by household income and caregiver education.

For ECDI scores, child sex, F(1, 344) = 7.555, *p* = 0.006, ηp^2^ = 0.021, child age, F(1, 344) = 10.800, *p* = 0.001, ηp^2^ = 0.030, household income, F(2, 344) = 5.335, *p* = 0.005, ηp^2^ = 0.030, and caregiver education, F(2, 344) = 8.728, *p* < 0.001, ηp^2^ = 0.048, were significant predictors. The Income × Caregiver Education interaction was also significant, F(4, 344) = 5.066, *p* < 0.001, ηp^2^ = 0.056, indicating that the association between household income and ECDI scores differed across caregiver education levels. Adjusted means, standard errors, and 95% confidence intervals are presented in Table 4, and the interaction pattern is displayed in Figure 1.

To further examine the significant Income × Caregiver Education interaction for ECDI scores, simple-effects analyses were conducted using estimated marginal means with Bonferroni-adjusted pairwise comparisons. Among children whose caregivers had less than a high school education, the low-income group had significantly higher adjusted ECDI scores than the middle-income group, mean difference = 0.274, SE = 0.128, *p* = 0.033, whereas the other income-group comparisons were not significant. Among children whose caregivers had a high school education, the low-income group had slightly higher adjusted ECDI scores than the high-income group, mean difference = 0.096, SE = 0.047, *p* = 0.042, while the other comparisons were not significant. Among children whose caregivers had more than a high school education, the high-income group had significantly higher adjusted ECDI scores than both the low-income group, mean difference = 0.150, SE = 0.067, *p* = 0.024, and the middle-income group, mean difference = 0.197, SE = 0.079, *p* = 0.013. These results indicate that income-related differences in ECDI scores varied across caregiver education levels, consistent with the significant interaction shown in Figure 1.

Table 4 presents the adjusted means, standard errors, and 95% confidence intervals for ICCE and ECDI scores across household income and caregiver education groups. These adjusted means correspond to the interaction pattern shown in Figure 1.

## 4. Discussion

The current study shows that poverty and caregiver education shape early childhood development in Indonesia through distinct yet related pathways. Financial capital and educational capital appeared to play distinct roles, which is consistent with socioeconomic models showing that household income and parental education may influence children through different family processes [11,20]. When the broad child-rearing environment was examined using the ICCE, both household income and caregiver education were significant predictors, but their interaction was not significant. This suggests that income and caregiver education may function as parallel and additive resources associated with the home environment. Higher income may make it easier for families to provide books, toys, nutritious food, safe spaces, and opportunities for children to participate in stimulating activities [25]. At the same time, caregiver education may support the home environment by shaping knowledge about child development, daily routines, communication practices, and non-harsh discipline [6,20]. Therefore, the present findings suggest that both material resources and caregiver knowledge contribute to children’s early experiences, although they may do so through different pathways [2,26].

The absence of an interaction effect for ICCE suggests that financial and educational resources were not substitutes for one another in shaping the child-rearing environment. This may reflect the mixed nature of the ICCE, which includes both material aspects of the home and caregiver-related practices. Income may be more relevant for material inputs, whereas caregiver education may be more closely related to caregiving knowledge, routines, and behavior. This interpretation is consistent with evidence that poverty affects early development through material hardship, parenting stress, and reduced opportunities for cognitive stimulation [27].

However, a different pattern was observed when children’s developmental milestones were examined. Among children aged 3 and 4 years, the interaction between household income and caregiver education was statistically significant. This finding is consistent with the proposed educational buffering hypothesis, suggesting that caregiver education may be associated with smaller income-related differences in developmental outcomes. Educational patterns have also been described in developmental research, where family resources, caregiving quality, and early learning opportunities can moderate the effects of socioeconomic adversity on young children’s outcomes [2,27]. Children in low-income households appeared to have more favorable developmental outcomes when their primary caregivers had higher educational attainment. This result does not mean that caregiver education eliminates the effects of poverty. Rather, it suggests that education may equip caregivers with knowledge, skills, and adaptive strategies to support children’s development under economic constraints. This interpretation is consistent with the nurturing care framework, which emphasizes that responsive caregiving, early learning, nutrition, health, and security are jointly important for early childhood development [26].

One plausible explanation is that educated caregivers may be better able to transform limited household resources into meaningful developmental experiences. For instance, a low-income household may have fewer books, toys, or formal early learning opportunities, but a caregiver with higher education may still provide cognitive stimulation through conversation, storytelling, counting, singing, naming objects, and asking questions during everyday routines. Previous research has shown that the quantity and quality of caregiver speech are associated with children’s early vocabulary and language development [28,29]. Maternal responsiveness has also been linked to children’s achievement of early language milestones [30]. Therefore, caregiver education may be particularly important in low-income households because it can influence how caregivers use ordinary daily activities as opportunities for interaction, learning, and emotional support.

This interpretation is also consistent with the concept of responsive caregiving. Developmental literature emphasizes that reciprocal caregiver–child interactions, sometimes described as “serve-and-return” interactions, may support early development by providing children with contingent, emotionally meaningful, and cognitively stimulating responses [5,31]. Responsive caregiving is also a central component of the nurturing care framework for early childhood development [26]. However, in the present study, caregiver–child interaction quality, language input, and home learning activities were not directly measured. Therefore, these interactional processes should be understood as plausible mechanisms rather than demonstrated pathways. Nevertheless, the buffering pattern observed in the data is consistent with the possibility that educated caregivers may provide more responsive communication, richer language input, and more structured support for children’s emerging developmental skills.

Caregiver education may also contribute to child development by improving caregivers’ ability to access, understand, and use health and parenting information. In Indonesia, community-based services such as Posyandu and parenting support programs such as Bina Keluarga Balita may provide information on child growth, nutrition, immunization, parenting, and developmental monitoring. More educated caregivers may be better able to interpret this information, communicate with service providers, and apply recommendations at home. This interpretation is consistent with broader evidence that caregiver knowledge, service access, and parenting support are important for improving early childhood outcomes in low- and middle-income countries [32,33,34]. In low-resource contexts, the ability to navigate available services may be an important form of human capital. Global early childhood frameworks also emphasize that family support, health services, nutrition, early learning, and responsive caregiving should be integrated to support disadvantaged children [26].

Taken together, these findings highlight the importance of distinguishing between material and relational dimensions of early childhood environments. Financial resources may be particularly relevant for material inputs, whereas caregiver education may be more closely related to caregiving knowledge, interaction quality, and developmental support. This distinction may help explain why income and caregiver education showed independent associations with ICCE scores, while their interaction was observed for ECDI scores.

These findings contribute to the literature on early childhood development in low- and middle-income countries by showing that caregiver education may be particularly relevant for understanding developmental differences among economically disadvantaged families. Previous studies have documented that poverty, malnutrition, inadequate stimulation, and limited access to services place young children at risk of failing to reach their developmental potential [2,27,35]. The present study extends this literature by suggesting that caregiver education may moderate the relationship between household income and developmental milestones in the Indonesian context. This interpretation is also aligned with evidence showing that parenting and responsive stimulation interventions can improve child development in disadvantaged settings [33,34]. However, because the present study did not test a parenting education intervention, these findings should not be interpreted as direct evidence that such interventions would be effective in this population. Rather, they identify caregiver education as a potentially important factor to consider in future intervention development and evaluation.

From a policy and intervention design perspective, the results suggest that strategies to support low-income families may need to address both financial and educational constraints. Economic assistance, nutrition support, access to early childhood education, and improvements in living conditions remain important because material deprivation limits the resources families can provide for children [2,25]. At the same time, the observed buffering role of caregiver education suggests that future programs could examine whether strengthening caregiver knowledge, responsive interaction, positive discipline, and developmental monitoring improves child outcomes among low-income households. In the Indonesian context, existing community platforms such as Posyandu, Bina Keluarga Balita, and early childhood education services may provide feasible channels for testing and delivering such approaches. However, these recommendations should be understood as implications for future research, program design, and intervention evaluation, rather than as direct evidence of the effectiveness of Posyandu-based parenting education and caregiver training. Future studies should therefore assess whether combining material support with caregiver-focused guidance produces stronger developmental benefits than either approach alone.

This study has several strengths, including a relatively large sample size and the use of validated assessment tools. The developmental analysis was also limited to children aged 3 and 4 years, which helped ensure that the developmental instrument was applied to the appropriate age group. However, several limitations should be considered. First, the cross-sectional design prevents causal inference. The observed interaction between income and caregiver education should therefore be interpreted as an association, not as proof that caregiver education causes better developmental outcomes among low-income children. Second, the use of non-probability sampling and the unknown response rate may limit the generalizability of the findings to the broader population of caregivers in Indonesia. Third, the study relied on caregiver-reported information about the home environment and children’s developmental milestones, which may be affected by recall bias or social desirability bias, especially for sensitive caregiving practices such as harsh discipline. Fourth, the study did not directly measure caregiver–child interaction quality, home language input, caregiver stress, executive function, neural development, or physiological stress regulation. As a result, explanations involving responsive interaction, cognitive stimulation, and stress buffering should be interpreted as theoretically plausible mechanisms rather than mechanisms directly tested in this study. Finally, this study cannot determine whether parenting education, community-based parenting programs, or economic support interventions would produce similar buffering effects. Such questions require intervention or longitudinal research designs.

Future research should use longitudinal and intervention-based designs to examine whether the moderating role of caregiver education persists as children transition into formal schooling. Longitudinal studies would be especially useful because early socioeconomic disadvantage can have cumulative effects across childhood, while supporting caregiving experiences may support later cognitive, language, and socioemotional outcomes [27]. Further studies should also include direct observations of caregiver–child interaction, measures of home learning activities, caregiver mental health, service utilization, and child developmental assessments across multiple domains. Intervention studies are also needed to test whether caregiver-focused community-based parenting support programs can improve developmental outcomes among low-income Indonesian children. Finally, because the present study used de-identified secondary data, the authors could not provide direct referral or follow-up support when caregiver responses suggested possible developmental concerns. Future prospective studies should consider including referral pathways to appropriate local health or early childhood services. Despite these limitations, the present study suggests that caregiver education may play an important role in supporting early childhood development among low-income households in Indonesia, while also underscoring that educational support for caregivers should complement, rather than replace, efforts to reduce household poverty.

## 5. Conclusions

Household income and caregiver education were independently associated with the child-rearing environment, while their interaction was associated with early childhood development. These findings are consistent with an educational buffering hypothesis but should be interpreted cautiously because of the cross-sectional design. Caregiver education may be a promising focus for future intervention design; however, longitudinal and intervention studies are needed to test whether parenting education, Posyandu-based support, or combined economic and caregiver-focused programs improve developmental outcomes in Indonesia.

## Figures and Tables

**Table 1:** Sociodemographic characteristics of the full sample and the 3–4-year-old subset.

Characteristic	Full Sample (*N* = 933)		Subset (*N* = 355)	
	*n*	%	*n*	%
Child Sex				
Girl	468	50.2	191	53.8
Boy	465	49.8	164	46.2
Child Age				
3 years old	81	8.7	81	22.8
4 years old	274	29.4	274	77.2
5 years old	444	47.6	-	-
6 years old	134	14.4	-	-
Household Monthly Income (IDR)				
Low (<3,000,000)	382	40.9	147	41.4
Middle (3,000,000–4,500,000)	157	16.8	56	15.8
High (>4,500,000)	394	42.2	152	42.8
Caregiver–child relationship				
Mother	878	94.1	325	91.5
Father	32	3.4	12	3.4
Other	23	2.5	18	5.1
Caregiver Education				
Less than high school	130	13.9	40	11.3
High school	475	50.9	167	47.0
More than high school	328	35.2	148	41.7

**Table 2:** Descriptive statistics for ICCE and ECDI by household income and caregiver education.

Predictor	*n*	ICCE, M (SD)	ECDI, M (SD)
Household monthly income (IDR)			
Low	382	10.17 (1.97)	3.81 (0.42)
Middle	157	10.53 (1.88)	3.74 (0.52)
High	394	10.82 (1.87)	3.80 (0.43)
Caregiver Education			
Less than high school	130	10.00 (2.15)	3.73 (0.51)
High school	475	10.31 (1.94)	3.81 (0.41)
More than high school	328	10.99 (1.73)	3.75 (0.46)

**Table 3:** General Linear Model results for the effects of household income, caregiver education, and their interaction on ICCE and ECDI scores.

Predictor	Child-Rearing				Early Childhood			
	Environment (ICCE)				Development (ECDI)			
	df	F	*p*-Value	ηp^2^	df	F	*p*-Value	ηp^2^
Child Sex (Covariate)	1, 922	0.006	0.937	0.000	1, 344	7.555	0.006	0.021
Child Age (Covariate)	1, 922	0.513	0.474	0.001	1, 344	10.800	0.001	0.030
Household Income	2, 922	3.192	0.042	0.007	2, 344	5.335	0.005	0.030
Caregiver Education	2, 922	3.877	0.021	0.008	2, 344	8.728	<0.001	0.048
Income × Education	4, 922	0.994	0.410	0.002	4, 344	5.066	<0.001	0.056

Notes: ICCE = Index of Child Care Environment; ECDI = Early Childhood Development Index. ICCE analyses used the full sample (*N* = 933), and ECDI analyses used the subset aged 3–4 years (*N* = 355). ηp^2^ = partial eta squared.

**Table 4:** Adjusted means, standard errors, and 95% confidence intervals for ECDI and ICCE scores by household income and caregiver education.

Household Income	Caregiver Education	ECDI				ICCE			
		M_adj_	*SE*	95% CI		M_adj_	*SE*	95% CI	
				LL	UL			LL	UL
Low	Less than high school	3.726	0.045	3.64	3.81	9.826	0.195	9.44	10.21
Low	High school	3.816	0.030	3.76	3.88	10.191	0.132	9.93	10.45
Low	More than high school	3.668	0.061	3.55	3.79	10.685	0.268	10.16	11.21
Middle	Less than high school	3.452	0.121	3.21	3.69	10.382	0.530	9.34	11.42
Middle	High school	3.770	0.043	3.69	3.86	10.490	0.190	10.12	10.86
Middle	More than high school	3.622	0.075	3.48	3.77	10.663	0.327	10.02	11.31
High	Less than high school	3.616	0.110	3.40	3.83	10.833	0.480	9.89	11.78
High	High school	3.721	0.039	3.65	3.80	10.333	0.169	10.00	10.66
High	More than high school	3.819	0.030	3.76	3.88	11.078	0.130	10.82	11.33

Notes: Madj = adjusted mean; SE = standard error; CI = confidence interval. Means were adjusted for child age and sex.

## Data Availability

The datasets generated and/or analyzed during the current study are not publicly available due to privacy and ethical restrictions.

## Abbreviations

The following abbreviations are used in this manuscript:

ICCE	Index of Child Care Environment
ECDI	Early Childhood Development Index
